# Regional brain volume differences between males with and without autism spectrum disorder are highly age-dependent

**DOI:** 10.1186/s13229-015-0022-3

**Published:** 2015-05-21

**Authors:** Hsiang-Yuan Lin, Hsing-Chang Ni, Meng-Chuan Lai, Wen-Yih Isaac Tseng, Susan Shur-Fen Gau

**Affiliations:** Department of Psychiatry, National Taiwan University Hospital and College of Medicine, No. 7 Chung-Shan South Road, Taipei, 10002 Taiwan; Graduate Institute of Clinical Medicine, National Taiwan University College of Medicine, No. 1, Jen-Ai Road Section 1, Taipei, 10051 Taiwan; Department of Child Psychiatry, Chang Gung Memorial Hospital at Linkou, No. 5 Fu-Hsing St., Taoyuan, 33305 Taiwan; Autism Research Centre, Department of Psychiatry, University of Cambridge, Douglas House, 18b Trumpington Road, Cambridge, CB2 8AH UK; Centre for Addiction and Mental Health, Hospital for Sick Children, and Department of Psychiatry, University of Toronto, 250 College St., Toronto, M5T 1R8 Canada; Graduate Institute of Brain and Mind Sciences, National Taiwan University College of Medicine, No. 1, Jen-Ai Road Section 1, Taipei, 10051 Taiwan; Center for Optoelectronic Medicine, National Taiwan University College of Medicine, No. 1, Jen-Ai Road Section 1, Taipei, 10051 Taiwan

**Keywords:** Autism spectrum disorder, Structural MRI, Age, Voxel-based morphometry, Regional brain volume, Development

## Abstract

**Background:**

Neuroanatomical differences between individuals with and without autism spectrum disorder (ASD) were inconsistent in the literature. Such heterogeneity may substantially originate from age-differential effects.

**Methods:**

Voxel-based morphometry was applied in 86 males with ASD and 90 typically developing control (TDC) males (aged 7 to 29 years). Three steps of statistical modeling (model 1, multiple regression with age as a covariate; model 2, multiple regression further considering diagnosis-by-age interaction; model 3, age-stratified analyses) were performed to dissect the moderating effects of age on diagnostic group differences in neuroanatomy.

**Results:**

Across ages, males with and without ASD did not differ significantly in total gray matter (GM) or white matter (WM) volumes. For both groups, total GM volumes decreased and WM volumes increased with age. For regional volume, comparing with the model only held the age constant (model 1), the main effect of group altered when diagnosis-by-age interaction effects were considered (model 2). Here, participants with ASD had significantly greater relative regional GM volumes than TDC in the right inferior orbitofrontal cortex and bilateral thalamus; for WM, participants with ASD were larger than TDC in the bilateral splenium of corpus callosum and right anterior corona radiata. Importantly, significant diagnosis-by-age interactions were identified at the bilateral anterior prefrontal cortex, bilateral cuneus, bilateral caudate, and the left cerebellum Crus I for GM and left forceps minor for WM. Finally, age-stratified analyses (model 3) showed distinct patterns in GM and WM volumetric alterations in ASD among subsamples of children, adolescents, and adults.

**Conclusions:**

Our findings suggest that the heterogeneous reports on the atypical neuroanatomy of ASD may substantially originate from age variation in the study samples. Age variation and its methodological and biological implications have to be carefully delineated in future studies of the neurobiology of ASD.

**Electronic supplementary material:**

The online version of this article (doi:10.1186/s13229-015-0022-3) contains supplementary material, which is available to authorized users.

## Background

Autism spectrum disorder (ASD) is a heterogeneous syndrome collectively characterized by early-onset difficulties in social communication and interactions and repetitive, restricted behaviors and interests [1,2]. Neuroimaging investigation has implicated structural abnormalities in several cortical and subcortical regions, yet no localized neuroanatomical features have been unambiguously identified [3]. Such inconsistency may be due to different methodologies or demographic heterogeneity [4], which may substantially affect the results of group comparisons, such as sex [5] and age [6]. As neural plasticity plays a crucial role in brain development across the life span, investigation into heterogeneity by age and age-dependent atypical neurobiology of ASD is pressingly needed.

Converging evidence across different studies points to the possibility that neuroanatomical differences between individuals with ASD and typically developing control (TDC) individuals are substantially age-dependent. Previous studies found that individuals with ASD have greater global [7] and lobar [8] gray/white matter volumes than TDC at early developmental stage (aged 2 to 8 years), yet without group differences later in childhood and teenage. In addition, regional brain volumetric differences between ASD and TDC are also age-dependent. For example, volumes of amygdala are larger in ASD than TDC in young childhood [9,10], comparable with TDC in late adolescence [11,12], but smaller than TDC in adulthood [13,14].

Global brain developmental trajectories between ASD and TDC may also be age-dependent. Longitudinal and cross-sectional data from participants aged 12 months to 50 years [15] demonstrate atypical neuroanatomical developmental trajectories of global volumes in ASD. Regionally, previous studies showed increased rate of amygdala enlargement in toddlers (aged 2 to 4 years) with ASD [16], whereas comparable rates of amygdala growth between ASD and TDC in early [17] and late [18] childhood. Moreover, diagnosis-by-age interactions in the cortical thickness of ‘social brain’ regions have been reported, but the results are inconsistent [19-23]. Non-linear age moderation effects on region-specific group differences have been further demonstrated by cross-sectional data [24]. Meta-analyses on published voxel-based morphometry (VBM) studies support age-dependent ASD-related characteristics in cortical and subcortical regions [6,25]. Lastly, longitudinal data also confirm region-specific group differences in growth trajectories [26,27]. The implication here is that when attempting to identify neurobiological characteristics of ASD in a cross-sectional study, it is crucial to consider age dependence, for instance by directly examining and controlling for diagnosis-by-age interaction effects and/or examining the main findings in different narrow-banded age-stratified groups.

However, to our knowledge, there have not been studies exploring how exactly the morphometric alterations for ASD may be obscured if age dependence is not formally modeled statistically. To dissect age moderation effects on diagnostic group differences in neuroanatomy with cross-sectional data, here, we adopted a three-step statistical modeling approach in a whole-brain VBM analysis. In model 1, we adopted a commonly used approach in past literature that investigated alterations in brain structure in individuals with and without ASD ‘controlled for’ the effect of age. The interpretation to the findings from this approach is about group differences when age is held constant. However, under this approach, it is unknown whether such main effect of diagnosis is directly interpretable, as the diagnosis-by-age interaction effect is not modeled and tested for. Therefore, one step further in model 2, we explicitly modeled the effect of diagnosis-by-age interaction. Given that previous studies have demonstrated potential different trajectories of brain development between individuals with and without ASD, we hypothesized that the diagnosis-by-age interaction effect would be evident in several brain regions. From the findings of model 2, the main effect of diagnosis is clearer and more interpretable, as (i) if regions showing main effects of diagnosis do not overlap with those showing diagnosis-by-age interaction, such main effects could be more reliably and directly interpreted, yet (ii) if regions showing main effects of diagnosis overlap with those showing diagnosis-by-age interaction, such main effects cannot be directly interpreted, and age-stratified analyses are indicated to disentangle the moderating effect of age on diagnostic effects. Besides, the comparison between the main effects identified in model 1 *versus* model 2 helps demonstrate how considering age-differential effects will alter the inferred main effects of diagnosis. Finally, the age-dependent main effect of diagnosis is further delineated in model 3 by age stratification (into child, adolescent, and adult subgroups) and by model selection to decide whether diagnosis-by-age interaction should be further modeled in these stratified, age-confined analyses. We hypothesized that the main effects of diagnosis would be different in different age ranges, and there would be no significant diagnosis-by-age interaction in model 3 owing to the more circumscribed age range. In this scenario, findings from model 3 will reveal the most interpretable diagnostic group differences in specific age bands, which at the same time clarifies how exactly age moderates diagnostic effects. Owing to the substantial heterogeneity in earlier reports of neuroanatomical studies in ASD [3-28], we did not hold specific hypothesis regarding specific regions showing significant effects in the abovementioned models.

## Methods

### Participants and procedure

The Research Ethics Committee at the National Taiwan University Hospital (NTUH) approved this study prior to implementation (9561709027, 200807036R, 201105115RC; ClinicalTrials.gov number, NCT00494754, NCT00755430, NCT01677793). The procedures and purpose of the study were explained face to face to the participants and their parents, who then provided written informed consent. All participants underwent the same clinical and MRI assessments, except that only the ASD group received the Chinese version of the Autism Diagnostic Interview-Revised (ADI-R) assessment.

We recruited 102 Taiwanese high-functioning males with ASD consecutively from the child psychiatry outpatient clinic of NTUH, and 90 TDC males from similar geographical districts (aged 7 to 29 years, full-scale IQ >70 in participants of both groups). Participants with ASD were clinically diagnosed according to the DSM-IV-TR and ICD-10 criteria by the corresponding author and further confirmed by interviewing the parents using the Chinese version of the ADI-R (translated into Mandarin and approved by Western Psychological Services) [29,30]. Thirteen participants with clinically diagnosed ASD did not meet the cut-off for ‘autism’ on the Chinese ADI-R and were thus excluded.

TDC participants were recruited if they did not have any current or lifetime DSM-IV psychiatric disorder based on the Chinese version of the K-SADS-E interview [31] and its modified adult version [32] with the participants and their parents. Exclusion criteria for both groups included past or current neurological or severe medical illness (for example, epilepsy), substance use disorders, schizophrenia, lifetime diagnoses of mood disorders, current anxiety disorders, and current use of psychotropic medication except methylphenidate (both immediate and extended release forms). The comorbidity and status of methylphenidate use in the ASD group (*n* = 86, participants in the final analyses following image quality control as indicated below) were described in Additional file 1: Table S1.

Intellectual function was assessed by the Wechsler Intelligence Scale for Children-3rd edition (WISC-III) [33] in participants aged 16 or younger or by the Wechsler Adult Intelligence Scale-Revised [34]. Handedness was assessed by the Edinburgh Inventory [35].

### Structural MRI acquisition and preprocessing

High-resolution T1-weighted images were acquired with a 3D magnetization prepared rapid acquisition gradient echo (MPRAGE) sequence on a 3 T MRI scanner (Siemens Magnetom Tim Trio, Munich, Germany) using a 32-channel phased arrayed head coil (parameters: TR = 2,000 ms; TE = 2.98 ms; TI = 900 ms; flip angle = 9°; FOV = 256 × 256 × 192 mm^3^; matrix size = 256 × 256 × 192; voxel size = 1 mm^3^ isotropic). Three ASD participants were excluded from further imaging processing owing to excessive in-scanner head motion by visual quality control inspection, yielding a final sample of 86 males with ASD and 90 TDC males.

Individual T1-weighted images were segmented by the New Segment toolbox in SPM8 (Wellcome Trust Centre for Neuroimaging, London, UK) to produce native space GM, WM, and cerebral spinal fluid (CSF) images. During segmentation, for all individuals below the age of 18 years, age- and sex-matched study-specific tissue probability maps generated from the Template-O-Matic toolbox (using the ‘matched-pair’ approach, matched for the distribution of age and sex with the present sample) were used; for individuals above 18 years old, default tissue probability map in New Segment was used. The native space GM and WM images of all participants (86 ASD, 90 TDC) were then registered to a study-specific template using a high-dimensional non-linear diffeomorphic registration algorithm (DARTEL) [36]. A modulation step was included to retain voxel-wise information about local tissue volume. The modulated GM and WM maps were smoothed with a 4-mm full-width at half-maximum Gaussian kernel. Individual total GM, WM, and CSF volumes were estimated by summing up the partial volume estimates throughout each class of segmented image in the native space. Total brain volumes were estimated by summing up total GM and WM volumes, whereas total intracranial volumes were calculated by summing up total GM, WM, and CSF volumes.

### Statistical analysis

The overview of analyses undertaken for brain volume was provided in Additional file 2: Table S2. Between-group differences in demographic data including age and IQ were examined using independent samples *t*-tests while handedness was examined by chi-square test (Table 1 and Additional file 3: Table S3, Additional file 4: Table S4 and Additional file 5: Table S5). Between-group differences in global brain volumes were examined using independent samples *t*-tests (Additional file 6: Table S6). Relationships between age and total GM, WM, and CSF volumes were demonstrated using Pearson’s correlation *r* (Additional file 7: Figure S1 for age distributions).

**Table 1:** **Demographics and clinical features**

	**ASD**	**TDC**	**Statistics**
	**(** ***n*** **= 86)**	**(** ***n*** **= 90)**	
Age, mean (SD)	15.0 (4.6)	15.7 (6.0)	*P* = 0.348
Handedness, right (%)	77 (89.5)	84 (93.3)	*P* = 0.367
Intelligence quotient (IQ)			
Full-scale IQ	102.9 (16.9)	114.4 (10.7)	*P* < 0.001
Verbal IQ	104.3 (17.3)	114.6 (9.6)	*P* < 0.001
Performance IQ	102.6 (17.4)	112.4 (12.8)	*P* < 0.001
Autism Diagnostic Interview-Revised			
Social	20.4 (5.5)	-	-
Communication	14.9 (4.6)	-	-
Repetitive and stereotyped behaviors	7.3 (2.7)	-	-

ASD, autism spectrum disorder; TDC, typically developing control; SD, standard deviation; IQ, intelligence quotient.

For regional neuroanatomy, voxel-wise mass univariate tests were performed in voxels included in tissue-specific templates, as indicated below, with SPM8. To avoid possible edge effects between different tissue types, the GM group comparisons were constrained within the GM segment of the study-specific template image with a threshold of partial volume estimates >0.25. A parallel procedure was processed for the WM group comparisons. Before statistical modeling, each modulated GM/WM map was rescaled in a tissue-specific manner, that is, GM and WM maps divided by individual total GM and WM volumes to derive a map indicating relative GM/WM volume, respectively.

#### Step 1: Are males with and without ASD, across a wide age range, different in regional neuroanatomy when the age effect was held constant?

In this first model, we fitted a general linear model at each voxel (*yi*), with the main effect of group (*Gi*) as a fixed factor and age (linear term) (*Ai*), full-scale IQ (FSIQ), and comorbidity status (Comorbidity) as nuisance covariates, to investigate between-group difference when the effect of age was held constant.

$$ \mathrm{Model}\;1:{\mathrm{y}}_{\mathrm{i}}={\upbeta}_0+{\upbeta}_1{\mathrm{G}}_{\mathrm{i}}+{\upbeta}_2{\mathrm{A}}_{\mathrm{i}}+{\upbeta}_3\mathrm{FSIQ}+{\upbeta}_4\mathrm{Comorbidity}+{\upvarepsilon}_{\mathrm{i}} $$

#### Step 2: Do regional neuroanatomical differences between males with and without ASD found in model 1 change when taking into consideration diagnosis-by-age interaction effects?

In this second model, we fitted a general linear model by further adding in an interaction term (G_i_*A_i_) to model 1, to test for diagnosis/group (G_i_) by age (A_i_) interaction effects. Significant interaction indicates that group differences in neuroanatomy are dependent on age.

$$ \mathrm{Model}\;2:\kern0.37em {\mathrm{y}}_{\mathrm{i}}={\upbeta}_0+{\upbeta}_1{\mathrm{G}}_{\mathrm{i}}+{\upbeta}_2{\mathrm{A}}_{\mathrm{i}}+{\upbeta}_3\left({\mathrm{G}}_{\mathrm{i}}*{\mathrm{A}}_{\mathrm{i}}\right)+{\upbeta}_4\mathrm{FSIQ}+{\upbeta}_5\mathrm{Comorbidity}+{\upvarepsilon}_{\mathrm{i}} $$

#### Step 3: Do regional neuroanatomical differences between males with and without ASD differ in age-stratified subgroups?

In the third model, we repeated model 2 and further stratified analyses by three age groups: children (7 to 12 years), adolescents (13 to 17 years), and young adults (18 to 29 years). The results of age-stratified analyses only partialling out age effects as done in model 1 were provided in Additional file 8: Table S7.

Age was mean-centered across all subjects before entering into all the models. We conducted all analyses with full-scale IQ (FSIQ) and the status of the comorbidity (a categorical/binary fixed-effect covariate) included as nuisance covariates (the results of statistical models without controlling for intelligence (though comorbidity status was still included as a nuisance covariate) were provided in Additional file 9: Table S8). Because the five participants with ASD who had taken methylphenidate were also among those who had psychiatric comorbidity, medication status was not added as an additional nuisance covariate to avoid overadjusting in the model. For VBM of all models, statistical outcomes were corrected for multiple comparisons at the cluster level by controlling topological family-wise error (FWE) calculated under Gaussian Random Field Theory, using a cluster-forming voxel-level height threshold of *P* < 0.005 and a spatial extent threshold (corrected for non-stationarity [4,37]) that ensures a cluster-wise FWE at *P* < 0.05. We localized GM structures using xjView toolbox (http://www.alivelearn.net/xjview). WM structures were labeled by overlaying the significant clusters with standard space WM tracts defined from JHU diffusion tensor imaging-based white matter atlases [38,39].

### Subsidiary analyses to confirm the main findings, with even and rectangular age distributions between groups

Considering the uneven age distribution between the two groups, and the fact that around 80% of participants with ASD aged within 10 to 19 years, which might result in unintended biases, we performed subsidiary analyses (using model 1 and model 2) by narrowing the age range of participants to 10- to 19-year-olds to ensure the age distribution of the two groups are even and rectangular (Additional file 10: Figure S2) for the age distributions of participants included in the subsidiary analyses; (Additional file 11: Table S9) for the demographic characteristics, as a re-examination of the main findings.

## Results

### Demographics

There were no significant differences between the ASD and TDC groups in age and handedness; however, TDC participants had significantly higher IQ profiles than ASD participants (Table 1) (Additional file 3: Table S3). For the three age-stratified ASD subgroups, there were no significant differences in handedness, IQ profile, and ADI-R subscores (Additional file 4: Table S4).

### Global brain volumes

**Figure 1:**
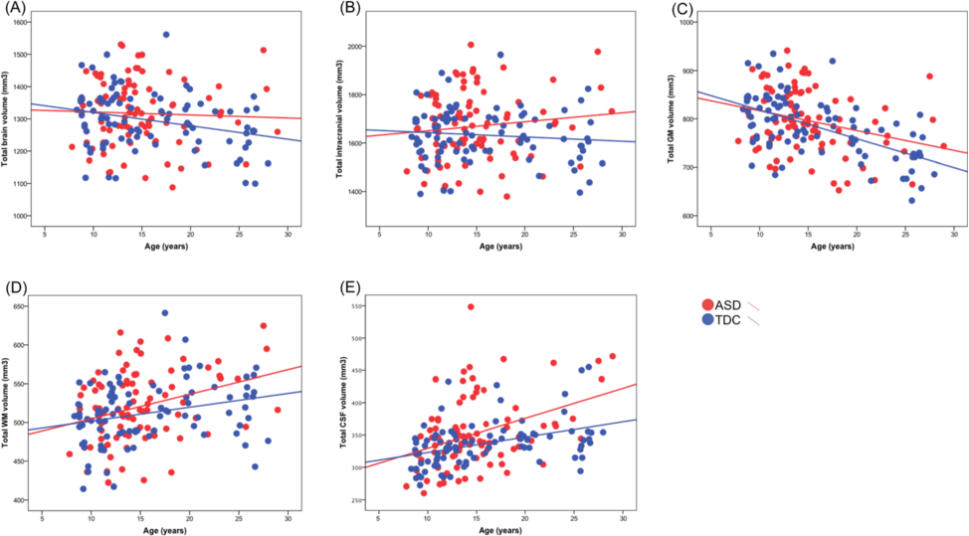
Correlations of **(A)** total brain, **(B)** intracranial, **(C)** total gray matter (GM), **(D)** total white matter (WM), **(E)** total cerebrospinal fluid (CSF) volumes with age in the autism spectrum disorder (ASD) and typically developing control (TDC) groups.

There were no significant group differences in intracranial, total brain, GM, or WM volumes between ASD and TDC, while participants with ASD had larger CSF volume than TDC (Additional file 6: Table S6). In both groups, total WM (ASD: *r* = 0.314, *P* = 0.003; TDC: *r* = 0.272, *P* = 0.009) and CSF (ASD: *r* = 0.384, *P* < 0.001; TDC: *r* = 0.406, *P* < 0.001) volumes increased significantly with age, whereas total GM volume decreased with age (ASD: *r* = −0.282, *P* = 0.009; TDC: *r* = −0.577, *P* < 0.001). Total brain volume decreased significantly with age in TDC (*r* = −0.280, *P* = 0.007) but was not associated with age in ASD (*r* = −0.041, *P* = 0.706). For intracranial volume, there was no significant age-volume correlation (ASD: *r* = 0.118, *P* = 0.279; TDC: *r* = −0.098, *P* = 0.356) (Figure 1). The group differences in age-volume correlational patterns did not reach statistical significance when using a multiple regression model with global brain volumes as dependent variables and diagnosis, age, and diagnosis-by-age interaction as regressors to test the effect of diagnosis-by-age interaction on global volumes. Finally, in the three age-stratified ASD subgroups, there were significant differences between age-subgroups in total GM, WM, and CSF volumes: there was no significant difference in total GM volume when directly comparing the child and adolescent subgroups, whereas total GM were significantly smaller in adults, relative to children and adolescents with ASD, respectively. In the age-stratified TDC subgroups, significant reduction of total GM volume was noted as age increased (that is, there were significant differences between child TDC and adolescent TDC and also between adolescent TDC and adult TDC subgroups). Total WM and CSF volumes increased with age in both ASD and TDC group (Additional file 4: Table S4, Additional file 5: Table S5).

### Regional neuroanatomical differences

#### Regional neuroanatomical differences: age effect held constant, without considering diagnosis-by-age interaction effects (model 1)

**Figure 2:**
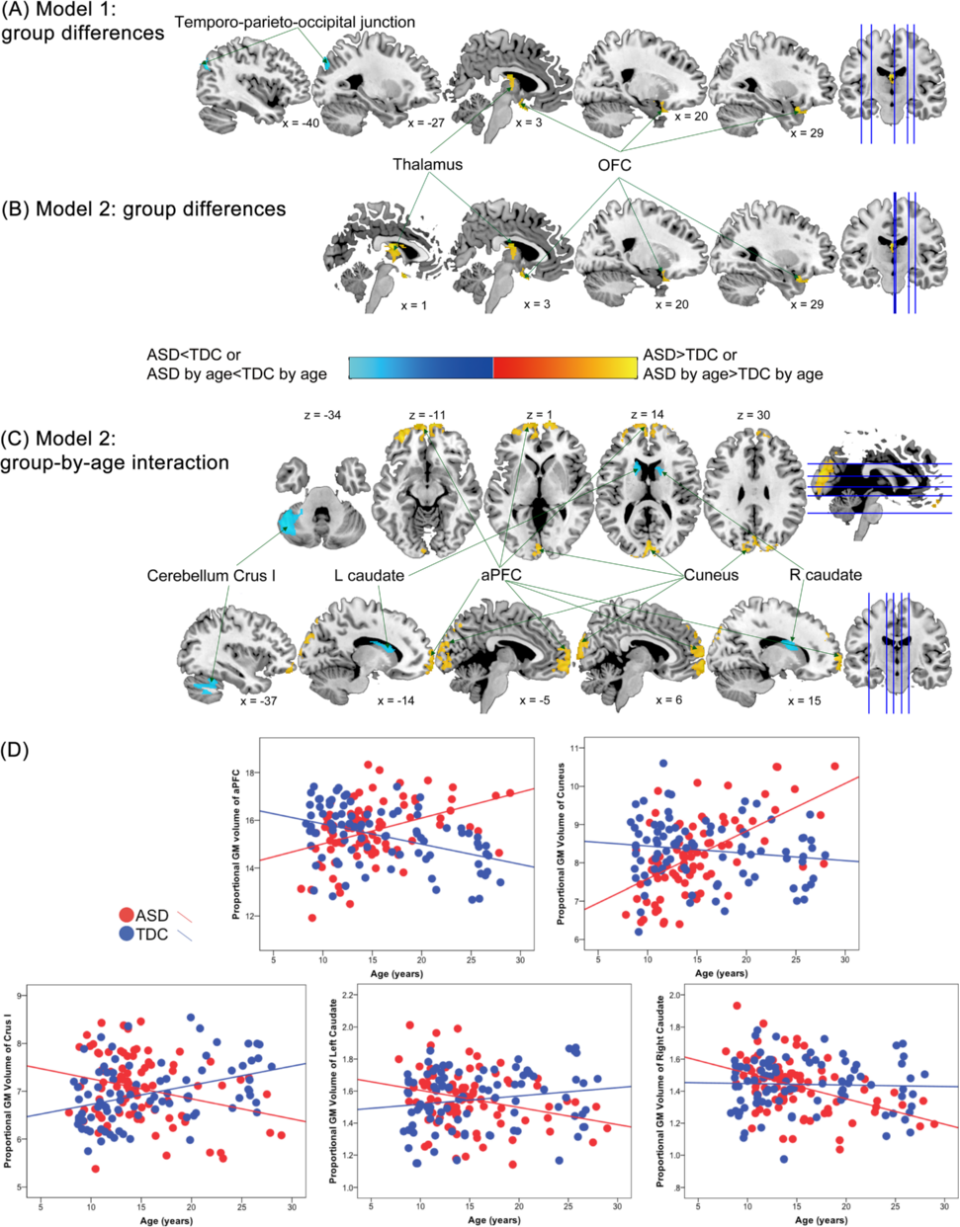
Group differences in relative regional gray matter (GM) volume between the autism spectrum disorder (ASD) and typically developing control (TDC) groups. **(A)** In model 1 (only controlling for age effects), individuals with ASD, relative to TDC, had larger relative GM volumes in the thalamus and orbitofrontal cortex (OFC) and smaller relative GM volumes in the temporo-parieto-occipital junction. **(B)** In model 2 (additionally considering diagnosis-by-age interaction effects), the main effect of diagnosis was identified in the thalamus and OFC (larger GM volumes in the ASD group, compared to TDC group), similar to those found in model 1. **(C)** The bilateral anterior prefrontal cortex (aPFC), bilateral cuneus, bilateral caudate, and left cerebellum Crus I showed significant diagnosis-by-age interaction in model 2. **(D)** Scatterplots descriptively illustrate the relationships between age and relative volumes of the regions showing significant diagnosis-by-age interaction.

**Figure 3:**
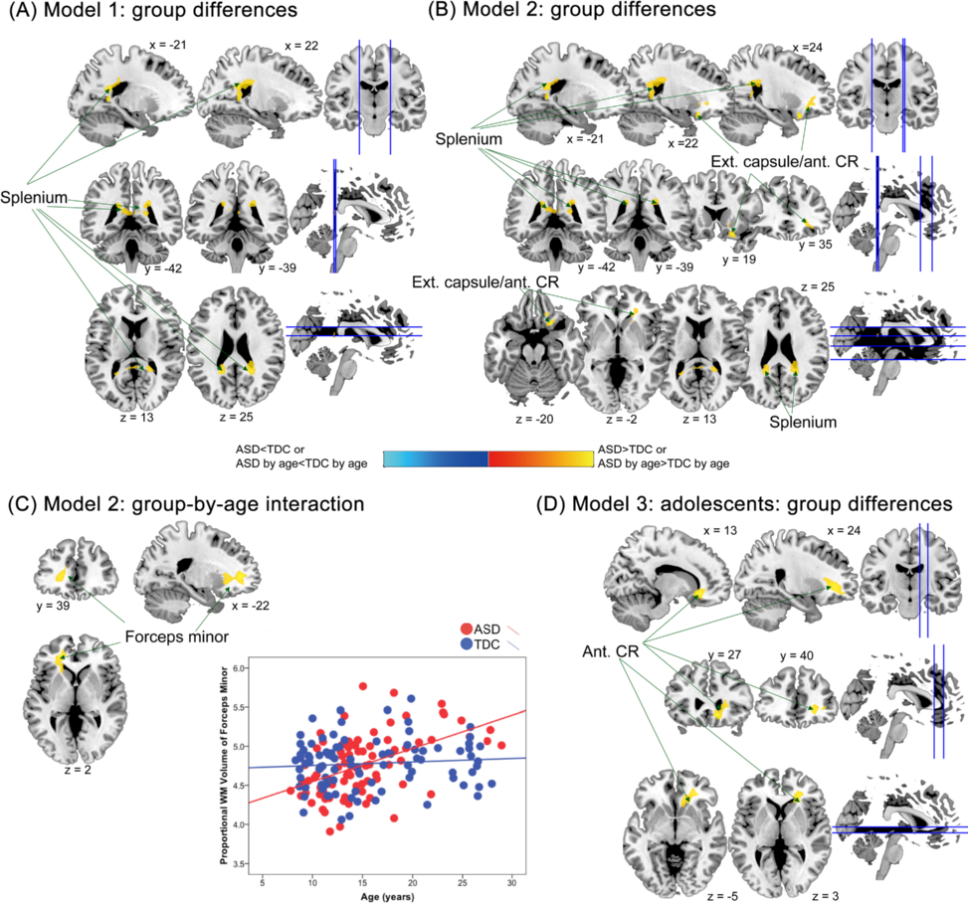
Group differences in relative regional white matter (WM) volume between the autism spectrum disorder (ASD) and typically developing control (TDC) groups. **(A)** In model 1 (only controlling for age effects), individuals with ASD, relative to TDC, had larger regional WM volumes in the splenium of corpus callosum. **(B)** In model 2 (additionally considering diagnosis-by-age interaction effects), there were larger WM volumes in the splenium and external capsule/anterior corona radiata (Ext. capsule/ant. CR), in ASD relative to TDC groups; **(C)** the left forceps minor showed significant diagnosis-by-age interaction in model 2, and its associated scatterplots for relationships between age and relative volumes of the region. **(D)** In model 3, adolescents with ASD had larger WM volumes in ant. CR, compared with TDC adolescents.

Results from model 1 showed that participants with ASD had significantly greater relative regional GM volumes in the right inferior orbitofrontal cortex and bilateral thalamus (Figure 2A, Table 2) but smaller relative regional GM volumes in the left temporo-parieto-occipital junction than TDC. For WM, participants with ASD had significantly greater relative regional WM volumes than TDC in the bilateral splenium of corpus callosum (Figure 3A, Table 3).

**Table 2:** **Significant differences in relative regional gray matter volume between participants with ASD and TDC participants, after controlling for full-scale IQ**

**Region**		**BA**	**Hemisphere**	**MNI coordinates**			***T*** **value**	**Cluster-level** ***P*** **value** ^**a**^	**Cluster size (voxels)**
				***x***	***y***	***z***			
Model 1: ASD > TDC									
Thalamus cluster	Medial dorsal nucleus of thalamus		L	−1	−12	19	4.71	0.014	563
	Medial dorsal nucleus of thalamus			0	−6	9	4.09		
	Lateral dorsal nucleus of thalamus		R	11	−21	15	3.71		
Right orbital frontal cluster	Inferior frontal gyrus	47	R	3	8	−24	4.68	0.002	761
			R	27	15	−23	4.37		
			R	30	27	−27	4.17		
Model 1: ASD < TDC									
Temporo-parieto-occipital junction cluster	Angular/middle occipital gyrus	39	L	−40	−75	30	3.81	0.058^b^	430
	Precuneus/middle occipital gyrus	19	L	−27	−84	40	3.75		
	Precuneus/middle occipital gyrus	19	L	−36	−75	42	3.51		
Model 2: ASD > TDC									
Thalamus cluster	Medial dorsal nucleus of thalamus		L	−1	−12	19	4.79	0.003	697
	Lateral dorsal nucleus of thalamus	…	R	11	−21	15	3.93		
			…	0	3	18	3.43		
Right inferior orbital frontal cluster	Inferior frontal gyrus	47	R	3	8	−24	4.63	0.009	605
	Inferior frontal gyrus	47	R	27	15	−23	4.35		
	Inferior frontal gyrus	47	R	30	27	−27	4.10		
Model 2: ASD by age > TDC by age									
Cuneus cluster	Cuneus	18		0	−90	16	5.51	<0.001	2,813
	Cuneus	19	R	26	−85	34	4.34		
	Cuneus	17	R	2	−82	12	4.31		
Anterior prefrontal cluster	Medial frontal gyrus	10	L	−6	71	−3	5.45	<0.001	4,277
	Superior frontal gyrus	10	L	−19	71	−3	5.27		
	Middle frontal gyrus	10	L	−30	51	−9	4.92		
Model 2: ASD by age < TDC by age									
Left cerebellum cluster	Crus I	…	L	−51	−58	−30	4.52	<0.001	1,666
	Crus I	…	L	−46	−48	−32	4.39		
	Crus I	…	L	−42	−42	−33	4.11		
Right caudate cluster	Caudate	…	R	18	3	16	4.01	0.050^b^	443
	Caudate	…	R	18	3	16	3.73		
	Caudate	…	R	12	−3	22	3.72		
Left caudate cluster	Caudate	…	L	−18	−1	24	3.57	0.042	457
	Caudate	…	L	−18	11	15	3.45		
	Caudate	…	L	−15	21	15	3.19		
Model 3: Child, ASD > TDC									
Limbic cluster	Subcallosal gyrus	34	L	−12	5	−15	3.91	0.002	732
	Sub-lobar	…	R	5	5	−12	3.90		
	Sub-lobar	…	L	−7	2	−8	3.80		
Model 3: Child, ASD < TDC									
Right postcentral cluster	Postcentral gyrus	3	R	41	−34	63	4.29	0.028	495
	Postcentral gyrus	3	R	35	−25	69	4.20		
	Postcentral gyrus	3	R	30	−34	64	4.06		
Left parieto-occipital junction cluster	Precuneus	19	L	−27	−82	39	4.19	0.016	546
	Precuneus	19	L	−34	−82	34	4.01		
	Middle occipital gyrus	19	L	−32	−91	24	3.43		
Model 3: Adult, ASD > TDC									
Right dorsal medial prefrontal cluster	Superior frontal gyrus	9	R	12	56	34	5.40	<0.001	1,192
	Superior frontal gyrus	10	R	8	51	42	4.89		
	Superior frontal gyrus	10	R	5	66	9	4.83		
Left anterior/dorsal medial prefrontal cluster	Superior frontal gyrus	10	L	−7	56	1	4.75	0.005	561
	Superior frontal gyrus	10	L	−7	72	−3	4.74		
	Medial frontal gyrus	10	L	−10	71	6	4.24		
Left lateral prefrontal cluster	Middle frontal gyrus	10	L	−39	51	−17	4.91	0.018	465
	Superior frontal gyrus	10	L	−33	59	−3	4.37		
	Superior frontal gyrus	10	L	−31	51	−8	3.82		
Cuneus cluster	Cuneus	18	R	2	−84	22	4.25	0.022	447
	Cuneus	18	…	0	−76	32	4.17		
	Cuneus	18	R	8	−91	14	4.11		

ASD, autism spectrum disorder; TDC, typically developing control; BA, Brodmann area; L, left; R, right; ellipses, not applicable; MNI, Montreal Neurological Institute. ^a^Statistical threshold was all set at FWE-corrected cluster-level *P* < 0.05, with cluster-forming voxel-level *P* < 0.005. ^b^Trend-level significant.

**Table 3:** **Significant differences in relative regional white matter volume between participants with ASD and TDC participants, after controlling for full-scale IQ**

**Tract-specific label**	**Nearest gray matter region**	**Hemisphere**	**MNI coordinates**			***T*** **value**	**Cluster-level** ***P*** **value** ^**a**^	**Cluster size (voxels)**
			***x***	***y***	***z***			
Model 1: ASD > TDC								
Splenium of corpus callosum	Precuneus	L	−18	−48	4	4.19	0.015	655
	Precuneus	L	−19	−46	21	3.57		
	Posterior cingulate gyrus	L	−16	−33	39	3.46		
Splenium of corpus callosum	Posterior cingulate gyrus	R	21	−46	6	3.65	0.009	715
	Precuneus	R	23	−46	24	3.46		
	Posterior cingulate gyrus	R	18	−30	30	3.27		
Model 2: ASD > TDC								
External capsule/anterior corona radiata	Inferior orbitofrontal cortex	R	23	20	−18	4.54	0.041	537
		R	26	32	−11	4.04		
		R	18	32	−18	3.64		
Splenium of corpus callosum	Precuneus	L	−18	−48	4	4.31	0.016	644
	Precuneus	L	−19	−46	21	3.61		
	Posterior cingulate gyrus	L	−16	−33	39	3.43		
Splenium of corpus callosum	Posterior cingulate gyrus	R	21	−46	7	3.60	0.011	690
	Precuneus	R	23	−46	24	3.42		
	Posterior cingulate gyrus	R	18	−30	30	3.30		
Model 2: ASD by age > TDC by age								
Forceps minor (anterior forceps)	Anterior cingulate gyrus	L	−18	45	4	4.14	0.001	960
		L	−25	20	−5	3.46		
		L	−18	29	−2	3.33		
Model 3: Adolescent, ASD > TDC								
Anterior corona radiata	Anterior cingulate gyrus	R	12	29	−9	4.10	0.001	1,151
		R	29	21	10	3.84		
		R	26	39	−5	3.74		

ASD, autism spectrum disorder; TDC, typically developing control; L, left; R, right; MNI, Montreal Neurological Institute. ^a^Statistical threshold was all set at FWE-corrected cluster-level *P* < 0.05, with cluster-forming voxel-level *P* < 0.005.

In the subsidiary analyses without controlling for full-scale IQ, smaller relative GM volumes of the left temporo-parieto-occipital junction in ASD, relative to TDC, remained significant. However, the results of greater GM volumes in ASD (relative to TDC) as identified in the main analysis were not significant anymore (Additional file 8: Table S7). There was no difference in regional WM volume between ASD and TDC groups.

#### Regional neuroanatomical differences: further modeling in diagnosis-by-age interaction effects (model 2)

Results in model 2 showed that participants with ASD had significantly greater relative regional GM volumes than TDC in the right inferior orbitofrontal cortex and bilateral thalamus (Figure 2B, Table 2), spatially overlapped with those found in model 1. Nonetheless, smaller volumes at the left temporo-parieto-occipital junction in ASD identified in model 1 were not found in model 2.

For WM, participants with ASD had significantly greater relative regional volumes than TDC in the bilateral splenium of corpus callosum (spatially overlapped with those found in model 1) and right anterior corona radiata (not identified as a main effect of diagnosis in model 1) (Figure 3B). No regions showed smaller relative regional WM volumes in ASD than TDC.

Importantly, model 2 identified significant diagnosis-by-age interaction effects in several regions including the GM of bilateral anterior PFC (aPFC/rostral prefrontal cortex, Brodmann area, BA, 10), bilateral cuneus (BA 17 to 19), left cerebellum Crus I, and bilateral caudate (Table 2, Figure 2C) and the WM of left forceps minor (Table 3, Figure 3C). Descriptively dissecting the interactions (Figure 2D), relative GM volume of the bilateral aPFC and cuneus increased with age in ASD but decreased in TDC, whereas relative GM volume of the left cerebellum Crus I decreased with age in ASD but increased in TDC. The volume of the bilateral caudate increased with age, and the volume of the forceps minor reduced with age in ASD, whereas there were no significant age-related changes in TDC.

In the statistical models without controlling for full-scale IQ, participants with ASD had significantly smaller relative regional GM volumes than TDC in the left temporo-parieto-occipital junction (Additional file 8: Table S7). Besides, diagnosis-by-age interaction remained significant in the bilateral aPFC, bilateral cuneus, and the left cerebellum Crus I (Additional file 8: Table S7). For WM, no group difference or diagnosis-by-age interaction effect was found when IQ was not controlled.

#### ASD-TDC difference in regional neuroanatomy, stratified by age (children, adolescents, and adults) (model 3)

**Figure 4:**
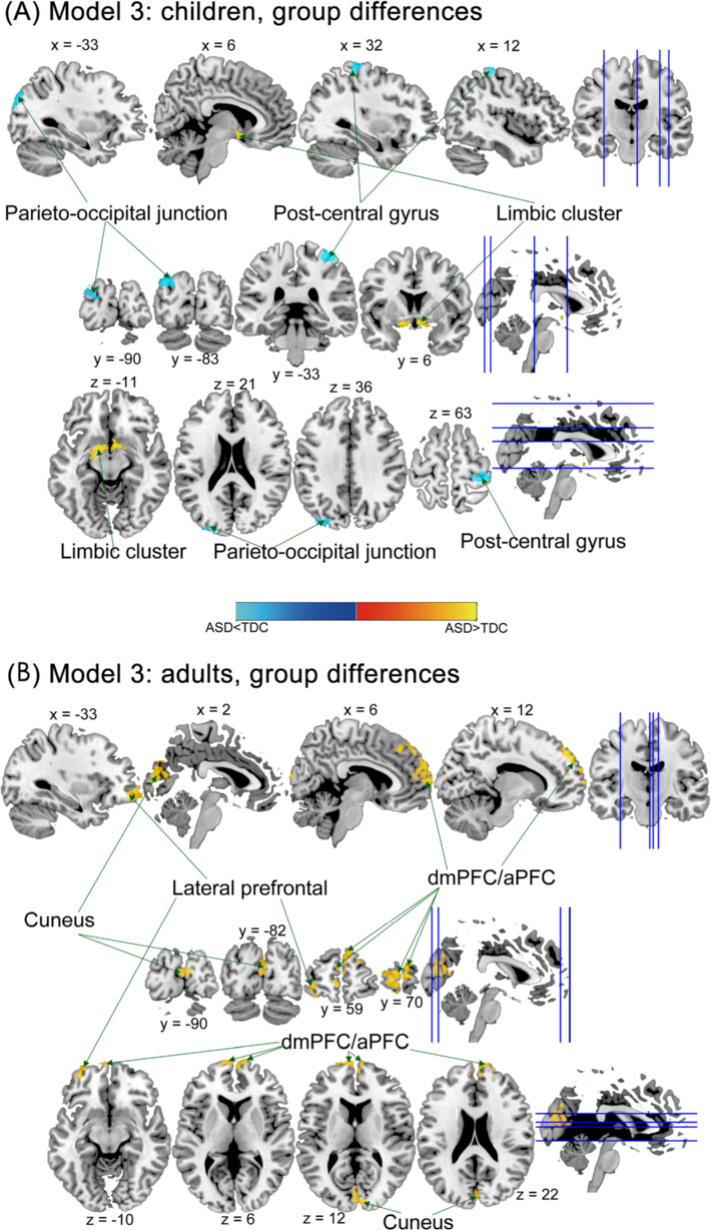
Group differences in relative regional gray matter (GM) volume between the autism spectrum disorder (ASD) and typically developing control (TDC) groups, stratified by children, adolescents (not shown), and adults (model 3). **(A)** Children with ASD had smaller GM volumes in the post-central gyrus and parieto-occipital junction and larger volumes in the limbic region, compared to TDC children; **(B)** in adult subgroup, there were larger volumes in the bilateral cuneus, bilateral dorsal medial prefrontal cortex/anterior prefrontal cortex (dmPFC/aPFC) in the ASD group relative to the TDC group.

First, children with ASD had greater relative regional GM volumes in the limbic region (including subcallosal gyrus and sublobar areas) but smaller GM volumes in the right post-central gyrus and left parieto-occipital junction than TDC children (Figure 4A, Table 2). However, in the statistical model without controlling for full-scale IQ, children with ASD had greater relative regional GM volumes in the limbic regions (subcallosal gyrus and extra-nuclear), but smaller GM volumes in bilateral aPFC (BA10) and the left cuneus (BA 18) (Additional file 8: Table S7). No significant group difference in regional WM volume was identified in the child subgroup, regardless of adjustment for IQ. There was no significant diagnosis-by-age interaction effect identified in the statistical models across all age subgroups. The spatial locations and extents of the main effects of diagnosis remained grossly the same across age-stratified analyses with (modeled as in model 2) or without (modeled as in model 1) considering diagnosis-by-age interaction effects (see Table 2 and Additional file 9: Table S8).

Second, in the adolescent subgroup, there were no significant group differences in relative regional GM volume, regardless of whether full-scale IQ effects were controlled for. However, in the statistical model controlling for IQ, adolescences with ASD had greater relative regional WM volumes in the right anterior corona radiata (Table 3).

Third, adults with ASD had greater relative regional GM volumes than TDC in the right dorsal mPFC (BA 9/10), left dorsal mPFC (BA10), left lateral frontal cortex (BA10), and right cuneus (BA 18) (Figure 4B, Table 2). In the statistical model without controlling for full-scale IQ, adults with ASD still had greater relative regional GM volumes than TDC in the right dorsal mPFC (BA 10), left lateral frontal cortex (BA10), and right cuneus (BA 18) (Additional file 8: Table S7). No significant group difference in regional WM volume was identified in the adult subgroup no matter controlling for IQ or not.

#### The subsidiary analyses in the restrained age range participants (with even and rectangular age distributions between the two groups)

In the subsidiary analyses using participants with restrained age range (10 to 19 years), model 1 identified that regional volume of the lingual gyrus was smaller in ASD relative to TDC, while this main effect of diagnosis was not found in model 2 (considering diagnosis-by-age interaction effects). Model 2 further identified significant diagnosis-by-age interaction effects, that relative GM volume of the bilateral cuneus and left cerebellar tonsil, alongside WM volume of the anterior limb of internal capsule, increased with age in ASD but decreased in TDC, whereas relative GM volume of the right superior temporal gyrus decreased with age in ASD but increased in TDC (Additional file 12: Table S10). Except for the bilateral cuneus clusters, other regions of GM and WM identified in the age-restrained subsidiary analyses, including the main effects of diagnosis and the interaction effects of diagnosis-by-age in model 1 and model 2, did not exactly overlap with those found in the main analyses in the whole sample.

## Discussion

In a large sample of males with or without ASD with age spanning 7 to 29 years, we found that statistically identified volumetric group differences altered when the diagnosis-by-age interaction effects were included in the model (by comparing results from model 2 to model 1). Using model 2, age dependency of atypical brain volume was identified in the GM of the bilateral anterior PFC, bilateral cuneus, left cerebellum crus I, and bilateral caudate, alongside the WM of the left forceps minor. Further stratification by age (in model 3) demonstrated substantial age-dependent regional brain volumetric differences between males with and without ASD. These findings were revealed after controlling for individual differences in tissue-specific brain volume, full-scale IQ, and comorbidity status. This three-step modeling approach adds evidence to support our speculation that the heterogeneity in morphometric alterations in ASD in the literature is substantially contributed by age variation in the samples. This suggests the necessity to account for age effects in neuroimaging research for ASD.

Our findings of negative age total GM volumes correlations in ASD were in accordance with an earlier cross-sectional report [40]. Nonetheless, the *post hoc* pair-wise comparisons demonstrated no significant differences between the child and adolescent subgroups. This might reflect an atypical developmental pattern (that is, late ‘normalization’) of total GM volume by adulthood in ASD, in comparison to the continuous GM volumetric reduction with age since early teenage found in typically developing individuals [41]. It also echoes findings of accelerated rate of decline in global brain size from adolescence to middle age in ASD [15]. At the level of regional neuroanatomy, our findings of diagnosis-by-age interaction effects were consistent with previous ASD studies showing significant age dependency in the diagnostic effects on regional volume [24,26,40] and cortical thickness [20-22,27], in particular the attenuation (or reversal) of the typical adolescence-to-adulthood linear decreased [41]. Explanations to these different age-dependent trends in both global and regional volumetric growth remain elusive. They may reflect processes of compensatory mechanisms, delayed maturation, or a phenomenon of ‘pseudonormalization’ (that is, there is a brief timespan in which brain volume appears ‘normal/typical’ in the course of atypical development [15]).

Regarding regional neuroanatomy, we posited that findings (for example, localization) of volumetric group differences might be different between using models with or without considering diagnosis-by-age interaction effects. The different findings from model 1 and model 2 supported this speculation. Based on this, we suggest that for any group comparison between individuals with and without ASD, it is best to first test for diagnosis-by-age interaction. In the modeling approach of the present study, only if the localization of group differences revealed in model 1 and model 2 overlap, that we are confident to say that diagnosis-by-age interaction is not producing substantial effects. The discrepant group difference findings between the two models suggest this is not the case in our sample; thus, it is a more appropriate modeling strategy to apply model 2 for main statistical inferences. Furthermore, when using model 2, if the localization of group effect and diagnosis-by-age effect overlap, the main effect of group becomes uninterpretable as how group difference presents itself is dependent upon the age of the individual. On the other hand, if the two does not overlap, both effects can be interpreted, separately. Under the latter circumstances (which is what we found), the noted diagnostic differences indicate regions where group differences are independent of age, and the noted regions showing diagnosis-by-age interaction effects indicate structures where group differences are substantially dependent on the age examined, potentially giving candidates for future longitudinal studies investigating group differential developmental trajectories. This view is also supported by a previous VBM meta-analysis [25] demonstrating that regions with significant ASD-TDC differences converge onto areas showing age-related dynamic brain growth in both groups. The mixed findings in prior VBM studies of ASD that only ‘controlled for’ age without considering diagnosis-by-age interaction effects [42-44] could therefore be understood in the light of the inconsistency regarding how age effects are treated.

Our results corroborate with previous reports on the age dependency of atypical brain growth patterns in ASD [16,20,21,24-27,40,45]. However, the directions of age-related regional brain volumetric changes for ASD and TDC appear to be mixed. Three methodological issues could complicate the interpretation and comparison of findings from the available literature: age span examined by the study, sample size of the study, and methods of data processing and statistical analysis. Here, we specifically contrast the present results with a recent large longitudinal study of brain volumetric growth in ASD [26]. Although Lange and colleagues’ study [26] and ours share similar features, such as investigating high-functioning male participants with ASD, having similar sample size, and examining similar age-range, age-related regional neuroanatomical change in ASD from these two studies appears discrepant. For example, our finding that the regional GM volume of the bilateral aPFC and cuneus increased with age in ASD but decreased in TDC is in contrary to the developmental trends of these compartmental volumes found in Lange *et al*. [26]. This inconsistency may arise partly from the different background of the samples (for example, ethnic/genetic influences or cultural influences on brain development) and from inferences made from different kinds of datasets, that is, cross-sectional data (revealing age effects from linear regression) *versus* longitudinal design (revealing true trajectories). In addition, different structural imaging protocols, that is, DARTEL under SPM8 in the current study *versus* surface-based analysis using FreeSurfer [46] in Lange *et al*. [26], may directly contribute to these disparities. VBM technique tends to find localized abnormalities, and the inference is based on locally averaged gray matter segmentation, which may be sensitive to inaccuracy of tissue classification and smoothing extents [47]. Surface-based method reconstructs cortical surface and provides direct measures of cortical morphology but may lack the sensitivity to detect subtle regional differences. These two techniques complement each other and may yield disparate results. Only one study to date combined these two methods to investigate neuroanatomy in ASD [42]. Using a large sample to directly compare and contrast the findings from different analysis protocols, such as VBM using SPM8 or FSL (FMRIB Software Library, www.fmrib.ox.ac.uk/fsl), surface-based morphometry using FreeSurfer, operator manual-tracing regional analyses, and so on, is still needed to disentangle the heterogeneous findings in autism neuroimaging [3,28], particularly in separating heterogeneity due to analytical methods from ‘true’ heterogeneity of the neurobiology of autism.

Moreover, as we applied a mass univariate analysis approach (VBM), only isolated locales with the largest group differences (or with large diagnosis-by-age interaction effects) would be revealed. However, this should not be taken to mean that our results reject the idea that abnormalities in multiple regions and neural systems characterize the neurobiology of autism. It is simply that the methodology applied here is less powerful in detecting systems-level atypicality, which will be more readily revealed by multivariate methodologies.

The subsidiary analyses using a confined-age group (10 to 19 years) identified similar age-dependent change in the cuneus region to the main finding, suggesting the robustness of such finding. However, disparity exists in other regions showing diagnosis-by-age interaction and main effect of diagnosis. Despite the relatively large sample size, we acknowledge that the small number of children (younger than 10 years old) and young adults (older than 20 years old) in the ASD group might introduce unintended bias from potential outliers in the diagnosis-by-age interaction analyses. These discrepancies in the regions demonstrating the main effect of diagnosis also further underscore that morphometric differences in ASD are largely dependent on the age window investigated. Future studies investigating age-related volumetric differences in ASD should investigate well-powered samples at younger and older ages. Otherwise, narrow-banded age-stratified analyses, as undertaken in model 3, on well-powered large samples, would provide a solution to this complex issue.

Following this argument, we posit that comparing groups in a wide age range sample without attempting to stratify by age may elude important developmental information. For example, in age-stratified analyses, we found age-specific diagnostic differences in GM of several brain regions. We also demonstrated that for the same region (for example, the cuneus and aPFC), ASD-TDC differences were opposite in direction between the child and adult age subgroups (in the analyses without controlling for full-scale IQ). A VBM meta-analysis [25] shows greater GM volumes in ASD than TDC over the right occipital lobe in younger age, but reduced GM volumes in older age, which comply with the age-specific patterns found in the present study.

Whether or not including IQ as a covariate in the model may also partially account for conflicting reports, as the present work showed that regional volumetric alteration in ASD did not exactly overlap between the statistical models with and without regressing out IQ effects. IQ is suggested to be related to the volume in several Brodmann areas, including BA 19 (corresponding to the cuneus) [48], and fluid intelligence is associated with the volume of mPFC in adults [49]. How IQ effects moderate noted atypical brain morphometry in ASD remains contentious. Some argue that low measured IQ is inherent in autism. Artificially ‘controlling for IQ effects’ would obscure the difference related to autistic symptoms [50] and might bring up confusion to the interpretation to the noted group differences [51]. The opposite view concerns that if IQ effects are not statistically controlled for, one may risk introducing type I errors that are related to intellectual differences rather than autism *per se* [52]. Additionally, prior evidence suggests that some age-related dynamic changes of cortical thickness in ASD may be modulated by intelligence [27]. Owing to these complex relationships, future investigation should also test for interactions between age, measured intelligence, and diagnosis.

In the present study, many regions showing atypical regional volume and age-dependent trends in ASD have been reported as major cortical and subcortical structures and white matter tracts implicated in the pathophysiology of ASD. The orbitofrontal cortex (associated with socio-emotional processing) exhibited atypical regional volume in ASD, consistent with prior studies [53,54]. Increased volume in thalamus (associated with sensory and cognitive processing [55]) in ASD is consistent with findings from a previous adult study [56]. Increased regional volume in anterior corona radiata, part of the limbic-thalamo-cortical circuitry [57], echoes the noted atypical white matter micro-structural integrity of this tract in ASD in previous studies [58,59]. Increased volume in ASD was found in the splenium of corpus callosum, inconsistent with prior MR morphometry studies [60-62]. Apart from different age ranges of sample and imaging methodology (as discussed earlier), possible reasons for the disparity could be underpowered samples in previous literatures and the non-linear relationship between corpus callosum and brain volume in the context of different correlations between brain volumes and IQ in ASD and TDC [63]. In any case, atypical volume of the splenium of corpus callosum, important for connecting posterior part of the emotional face processing domain [64] and default mode networks [65] can be associated with the idea of inter-hemispheric dysconnectivity in ASD [58,66,67].

The aPFC, caudate nucleus, and cerebellum Crus I, alongside forceps minor, represent the major cortical, subcortical hubs, and white matter tract of the cognitive control network [68,69]. Besides, the cuneus involves visual processing network. These structures all demonstrated atypical age-dependent trends in ASD. Differential age-dependent changes of caudate volume are concordant with earlier reports [40,45]. Differences in the cuneus also echo a recent longitudinal study [27] suggesting greater age-related cortical thinning in the cuneus in ASD. However, the direction of age-related changes is opposite from our findings, which may be partly driven by differences in aspects of neuroanatomy examined (cortical thickness *versus* volume) as well as other methodological issues discussed earlier. Other regions (that is, the aPFC, cerebellum Crus I, and forceps minor) demonstrating atypical age-dependent trends are first reported in the present study.

The aPFC has been suggested to contribute to a range of higher-order cognitive functions, including multitasking [70], memory retrieval [71], mentalizing [72,73], and joint attention [74,75], all critically associated with ASD [76-78]. Furthermore, adults with high-functioning autism have atypical morphometry [79,80], functional specification during mentalizing and attention [81], and cerebral blood flow [82] at the aPFC. Interestingly, one recent fMRI study [83] shows a similar age-dependent pattern of neural activation over the right mPFC (negatively associated with age in TDC, positively associated with age in ASD) during explicit empathy task, suggesting possible convergence of age-dependent changes in anatomy and function at the aPFC in ASD. The forceps minor comprises homotopic fibers that communicate between the bilateral aPFC. The concordance in atypical age-dependent growth of volume of the aPFC and forceps minor complies with a recent meta-analysis [84] showing a considerable degree of convergence among GM and WM morphometric alterations in the frontal regions in ASD. This positive GM/WM concordance may be explained by the tropic effect [85], that is, direct axonal connections result in cortical thickness change in the same direction. Taken together, these findings suggest the indispensable role of the prefrontal cortex (especially BA 10) in the dynamic processes underpinning the developmental pathophysiology of ASD.

A growing body of evidence indicates that the cerebellum is involved in the pathophysiology of ASD [86,87], in relation to executive control and social cognition. There are few prior studies directly examining and reporting age effects on cerebellar structures in ASD. To our knowledge, we are the first to report age dependency in diagnostic volumetric changes in the left Crus I, and the directions of age-related changes in TDC concur with typical morphometric development in childhood and adolescence [88]. In individuals with ASD, decreased GM volume is consistently found in the right Crus I across different age ranges [89,90] and is associated with repetitive and stereotyped behaviors [44]. In non-ASD individuals, those who are homozygous for an allele on the autism candidate gene CNTNAP2 show significantly reduced GM in the cerebellum in bilateral Crus I [91]. Reduced functional connectivity between the right Crus I and fronto-parietal network [92] and lower Purkinje cell density in bilateral Crus I [93] have also been reported in ASD. All the evidence converges to suggest that differences in the Crus I may contribute to the pathophysiology of ASD. Intriguingly, in the subsidiary age-constrained analyses, diagnosis-by-age interaction was identified in the left cerebellar tonsil with the opposite age-dependent trend with the Crus I, that is, smaller volumes in younger ASD and larger volumes in older ASD. This pattern is in line with a report suggesting larger cerebellar tonsil volumes in adults with ASD [94] and corroborates abnormal GM volume of the region identified in an activation likelihood estimation meta-analysis [89]. In TDC, these two cerebellar regions functionally connect with the prefrontal regions in a distinct way (Crus I with the dorsolateral prefrontal cortex in the cognitive control system; tonsils with the motor cortex in the motor control network) [95]. The separate age-dependent trends in ASD echo earlier reports of region-specific patterns of cerebellar anatomical alterations in ASD [90,96]. How atypical volume of these cerebellar sub-regions relates to functional abnormalities in ASD requires further investigation.

The brain structural differences in ASD might be the product of atypical neurogenesis/gliogenesis, neuronal cell shrinkage/death, synaptic pruning, or myelination. Consistent with the present findings, postmortem studies on the neuropathology of ASD demonstrate altered cytoarchitectural organization [97], perturbed size and density of minicolumns [98], and increased microglial infiltration [99,100] in the regions identified here. These heterogeneous microstructural changes in neurobiology may add up to macrostructural differences in ASD as shown in the present study (see [101] for a review).

Overall, our findings in the context of the biological, methodological, and clinical heterogeneity of ASD raise several questions to be further addressed: What drives the atypical developmental trajectories of brain growth in individuals with ASD compared to typically developing individuals? How are these neurobiological atypicalities associated with the developmental changes in cognition and behavior?

### Limitations

Our findings should be interpreted in the light of other limitations not indicated above. First, the cross-sectional design limits the capacity in directly addressing developmental trajectories, for example, any non-linear trends. The age dependency shown in this work should be interpreted in combination with findings from longitudinal studies [26,27], given the inherent weakness of cross-sectional data in drawing inferences about longitudinal processes. Second, as the participants were of average or above-average IQ and were all males, it is unknown whether the findings could be generalized to other demographic subgroups [3,4]. Third, there were five males with ASD taking methylphenidate for inattentive symptoms. Despite a lack of information regarding methylphenidate effects on brain structures in ASD, prior literature provides evidence that stimulant treatment may normalize some structural brain abnormalities in attention-deficit/hyperactivity disorder [102]. How exactly stimulant treatment affects brain structures in ASD remains to be clarified. Lastly, the age-stratified analysis was based on arbitrary chronological age cutoffs owing to the lack of pubertal development information in our study. Prior studies suggest that hormonal processes around puberty exert significant effects on brain structural development [103,104], warranting further inquiry into structural brain alterations in ASD in relation to physical and hormonal developmental stages.

## Conclusions

In summary, our three-step statistical modeling approach demonstrated highly age-dependent atypical brain morphometry in ASD. Diagnosis-by-age interaction should be regularly examined, and age-stratified analyses should be further performed in future studies into the developmental neurobiology of ASD. Our findings, together with comparisons with earlier reports, indicate that prior discrepant structural neuroimaging findings in ASD may substantially originate from the various age range examined, as well as different statistical approach dealing with age dependency effects. Besides, different imaging protocols and the biological heterogeneity of ASD could further contribute to the discrepancy in the literature. Longitudinal studies with more even age distributions and better-powered designs, alongside the conjoint use of several imaging analysis pipelines can help directly clarify plausible differential growth trajectories in the life span neurodevelopment in ASD.

## Additional files

Additional file 1: Table S1. Comorbidity and medication use in individuals with autism spectrum disorder. Additional file 2: Table S2. Overview of analyses undertaken for brain volume. Additional file 3: Table S3. Demographics of participants stratified by age group. Additional file 4: Table S4. Comparisons of demographics, IQ profiles, ASD symptoms, and brain volumes among three age-stratified subgroups in the autism spectrum disorder group. Additional file 5: Table S5. Comparisons of demographics, IQ profiles, and brain volumes among three age-stratified subgroups in typically developing controls. Additional file 6: Table S6. Global brain volume differences. Additional file 7: Figure S1. Age distributions of participants with autism spectrum disorder and typically developing controls of the main analyses. Additional file 8: Table S7. Significant differences in relative regional gray matter volume between participants with ASD and TDC participants, without controlling for full-scale IQ. Additional file 9: Table S8. Significant differences in relative regional gray matter and white matter volume between participants with ASD and TDC participants in age-stratified analyses (model 3), only partialling out age effects as done in model 1. Additional file 10: Figure S2. Age distributions of participants with autism spectrum disorder and typically developing controls of the secondary analyses for the restrained age range. Additional file 11: Table S9. Demographics of participants of restrained age range. Additional file 12: Table S10. Significant differences in relative regional volume between participants with ASD and TDC participants of restrained age range.

## Abbreviations

ADI-R: Autism Diagnostic Interview-Revised
aPFC: anterior prefrontal cortex
ASD: autism spectrum disorder
BA: Brodmann area
CNTNAP2: contactin-associated protein-like 2
DARTEL: diffeomorphic anatomical registration using exponentiated Lie algebra
DSM-IV: Diagnostic and Statistical Manual of Mental Disorders-Fourth Edition
fMRI: functional magnetic resonance imaging
FOV: field of view
FWE: family-wise error
GM: gray matter
IQ: intelligence quotient
mPFC: medial prefrontal cortex
MPRAGE: magnetization prepared rapid acquisition gradient echo
MRI: magnetic resonance imaging
NTUH: National Taiwan University Hospital
SPM: statistical parametric mapping
TD: typically developing
TDC: typically developing controls
TE: echo time
TI: inversion time
TR: repetition time
VBM: voxel-based morphometry
WISC-III: Wechsler Intelligence Scale for Children-Third Edition
WM: white matter
