# Supplementary material for: Regional brain volume differences between males with and without autism spectrum disorder are highly age-dependent
Source: Mol Autism. 2015 May 21;6:29. doi: 10.1186/s13229-015-0022-3 (PMC4455336; doi:10.1186/s13229-015-0022-3)
Supplement: Additional file 1: Table S1. — Comorbidity and medication use in individuals with autism spectrum disorder. [file 13229_2015_22_MOESM1_ESM.pdf]

Additional file 1: Table S1 Comorbidity and medication use in individuals with autism spectrum disorder (ASD)

Diagnosis	N	Psychostimulant[a] use (N)	Other medication use (N)
**ASD in total**	86	5	0
**ASD only**	73	0	0
**Co-occurring ADHD only**	7	4	0
**Co-occurring learning disorders only**	3	0	0
**Co-occurring tic disorder only**[c]	1	0	0
**Co-occurring ADHD and tic disorder**[c]	1	1	0
**Co-occurring learning disorder and tic disorder**[d]	1	0	0

ADHD, attention-deficit/hyperactivity disorder
[a]Only methylphenidate (both immediate-release and extended-release) was prescribeded.
[b]One had co-occurring writing disorder, the other two had both reading and writing disorders.
[c]Motor tic disorder.
[d]Co-occurring reading and motor tic disorder.
