# Supplementary material for: Regional brain volume differences between males with and without autism spectrum disorder are highly age-dependent
Source: Mol Autism. 2015 May 21;6:29. doi: 10.1186/s13229-015-0022-3 (PMC4455336; doi:10.1186/s13229-015-0022-3)
Supplement: Additional file 2: Table S2. — Overview of analyses undertaken for brain volume. [file 13229_2015_22_MOESM2_ESM.pdf]

Additional file 2: Table S2 Overview of analyses undertaken for brain volume

- **Global brain volumes**
  - Group differences in total intracranial, total brain, total gray matter, total white matter, alongside total cerebrospinal fluid volumes between participants with ASD and TDC (*Additional Table 6*); between age subgroups in the ASD group (*Additional Table 4*); between age subgroups in the TDC group (*Additional Table 5*).
  - Correlations between age and global brain volumes in both ASD and TDC groups (*Figure 1*).
- **Regional brain volumes of gray matter and white matter**
  - **<u>Main analyses</u>**, full-scale intelligence quotient and comorbidity status included as nuisance covariates (*Table 2 & 3; Figure 2-4*)
    - Step (Model) 1: Regional neuroanatomical differences: age effect held constant, without considering diagnosis-by-age interaction effects
    - Step (Model) 2: Regional neuroanatomical differences: further modeling in diagnosis-by-age interaction effects
    - Step (Model) 3: ASD-TDC difference in regional neuroanatomy, stratified by age (children, adolescents, and adults),
      - Statistical model as Model 2 (*Table 2 & 3; Figure 3*)
      - Statistical model as Model 1 (*Additional Table 7*)
  - **<u>Subsidiary analyses 1</u>**: Model 1-3 undertaken without controlling for intelligence, whereas comorbidity status still included as a nuisance covariate (*Additional Table 8*).
  - **<u>Subsidiary analyses 2</u>**: Model 1 and 2 in the restrained age-range participants (age 10-19 years), with even and rectangular age distributions between the two groups (*Additional Table 10*).

Abbreviation: ASD, autism spectrum disorder; TDC, typically developing control
