# Supplementary material for: Regional brain volume differences between males with and without autism spectrum disorder are highly age-dependent
Source: Mol Autism. 2015 May 21;6:29. doi: 10.1186/s13229-015-0022-3 (PMC4455336; doi:10.1186/s13229-015-0022-3)
Supplement: Additional file 3: Table S3. — Demographics of participants stratified by age group. [file 13229_2015_22_MOESM3_ESM.pdf]

Additional file 3: Table S3 Demographics of participants stratified by age group

	ASD	TDC	Overall	Statistics
**Child: age of 7-12 years old**				
**Case numbers**	28	43	71	
**Age, mean (SD)**	10.7 (1.2)	10.6 (1.4)	10.7 (1.3)	p = 0.806
**Handedness, right (%)**	24 (85.7)	40 (93.0)	-	p = 0.313
**Intelligence Quotient (IQ)**				
Full-scale IQ	106.9 (17.9)	115.2 (10.0)	-	p = 0.014
Verbal IQ	107.0 (18.8)	115.4 (9.3)	-	p = 0.015
Performance IQ	106.4 (16.4)	112.6 (12.0)	-	p = 0.072
**Total gray matter volume (mm^3)**	807.6 (66.9)	814.2 (55.7)	811.6 (60.0)	p = 0.651
**Total white matter volume (mm^3)**	502.0 (42.6)	501.9 (37.5)	501.9 (39.3)	p = 0.997
**Total CSF volume (mm^3)**	325.0 (40.5)	324.6 (30.9)	324.8 (35.0)	p = 0.960
**Total brain volume (mm^3)**	1309.5 (106.6)	1316.2 (90.9)	1313.6 (96.7)	p = 0.780
**Total intracranial volume (mm^3)**	1634.6 (135.3)	1640.8 (113.3)	1638.3 (121.5)	p = 0.836
**Adolescent: age of 13-17 years old**				
**Case numbers**	40	18	58	
**Age, mean (SD)**	14.7 (1.3)	15.5 (1.6)	14.9 (1.4)	p = 0.043
**Handedness, right (%)**	37 (92.5)	17 (94.4)	-	p = 0.787
**Intelligence Quotient (IQ)**				
Full-scale IQ	101.5 (15.6)	108.7 (11.4)	-	p = 0.085
Verbal IQ	101.9 (16.4)	110.2 (9.1)	-	p = 0.050
Performance IQ	101.5 (17.8)	105.8 (13.7)	-	p = 0.369
**Total gray matter volume (mm^3)**	808.6 (59.0)	793.3 (43.3)	803.9 (54.7)	p = 0.330
**Total white matter volume (mm^3)**	525.3 (44.1)	510.7 (40.6)	520.8 (43.2)	p = 0.238
**Total CSF volume (mm^3)**	362.3 (58.6)	341.3 (34.2)	355.8 (52.8)	p = 0.164
**Total brain volume (mm^3)**	1333.9 (98.0)	1304.1 (82.3)	1324.7 (93.7)	p = 0.265
**Total intracranial volume (mm^3)**	1696.2 (143.2)	1645.4 (103.7)	1680.4 (133.4)	p = 0.182
**Adult: age of 18-29 years old**				
**Case numbers**	18	29	47	
**Age, mean (SD)**	22.2 (3.6)	23.4 (3.0)	22.9 (3.2)	p = 0.225
**Handedness, right (%)**	16 (88.9)	26 (89.7)	-	p = 0.934
**Intelligence Quotient (IQ)**				
Full-scale IQ	99.6 (17.7)	116.8 (10.2)	-	p < 0.001
Verbal IQ	105.3 (17.0)	116.1 (9.6)	-	p = 0.011
Performance IQ	98.1 (18.1)	116.4 (11.9)	-	p < 0.001
**Total gray matter volume (mm^3)**	751.8 (65.7)	734.2 (47.3)	740.9 (55.1)	p = 0.292
**Total white matter volume (mm^3)**	538.2 (47.7)	527.5 (37.0)	531.6 (41.2)	p = 0.397
**Total CSF volume (mm^3)**	373.3 (53.3)	351.2 (36.1)	360.0 (44.3)	p = 0.096
**Total brain volume (mm^3)**	1289.9 (111.4)	1261.7 (81.4)	1272.5 (94.0)	p = 0.322

**Total intracranial volume (mm^3)**	1663.3 (149.9)	1612.9 (103.5)	1632.2 (124.3)	p = 0.180

ASD, autism spectrum disorder; TDC, typically developing controls; CSF, cerebrospinal fluid; SD, standard deviation.
