# Supplementary material for: Regional brain volume differences between males with and without autism spectrum disorder are highly age-dependent
Source: Mol Autism. 2015 May 21;6:29. doi: 10.1186/s13229-015-0022-3 (PMC4455336; doi:10.1186/s13229-015-0022-3)
Supplement: Additional file 4: Table S4. — Comparisons of demographics, IQ profiles, ASD symptoms, and brain volumes among three age-stratified subgroups in the autism spectrum disorder group. [file 13229_2015_22_MOESM4_ESM.pdf]

Additional file 4: Table S4 Comparisons of demographics, IQ profiles, ASD Symptoms, and brain volumes among three age-stratified subgroups in the autism spectrum disorder group

Mean (SD)	Child	Adolescent	Adult	Statistics
**Age range**	7-12	13-17	18-29	-
**Age, mean (SD)**	10.7 (1.2)	14.7 (1.3)	22.2 (3.6)	-
**Handedness, right (%)**	24 (85.7)	37 (92.5)	16 (88.9)	p = 0.664
**Intelligence Quotient (IQ)**				
Full-scale IQ	106.9 (17.9)	101.5 (15.6)	99.6 (17.7)	p = 0.291
Verbal IQ	107.0 (18.8)	101.9 (16.4)	105.3 (17.0)	p = 0.477
Performance IQ	106.4 (16.4)	101.5 (17.8)	98.1 (18.1)	p = 0.290
**ADI-R, Diagnostic**				
Social	21.6 (5.3)	19.4 (5.5)	20.6 (5.4)	p = 0.255
Communication	15.4 (4.3)	14.8 (4.6)	14.3 (5.3)	p = 0.762
Behavior	7.6 (2.6)	7.6 (2.7)	6.2 (2.8)	p = 0.168
**Total gray matter volume (mm^3)**	807.6 (66.9)	808.6 (59.0)	751.8 (65.7)	p = 0.005
**Total white matter volume (mm^3)**	502.0 (42.6)	525.3 (44.1)	538.2 (47.7)	p = 0.020
**Total CSF volume (mm^3)**	325.0 (40.5)	362.3 (58.6)	373.3 (53.3)	p = 0.004
**Total brain volume (mm^3)**	1309.5 (106.6)	1333.9 (98.0)	1289.9 (111.4)	p = 0.301
**Total intracranial volume (mm^3)**	1634.6 (135.3)	1696.2 (143.2)	1663.3 (149.9)	p = 0.214

ASD, autism spectrum disorder; ADI-R, the autism diagnostic interview-revised; SD, standard deviation; CSF, cerebrospinal fluid; SD, standard deviation.
