# Supplementary material for: Regional brain volume differences between males with and without autism spectrum disorder are highly age-dependent
Source: Mol Autism. 2015 May 21;6:29. doi: 10.1186/s13229-015-0022-3 (PMC4455336; doi:10.1186/s13229-015-0022-3)
Supplement: Additional file 5: Table S5. — Comparisons of demographics, IQ profiles, and brain volumes among three age-stratified subgroups in typically developing controls. [file 13229_2015_22_MOESM5_ESM.pdf]

Additional file 5: Table S5 Comparisons of demographics, IQ profiles, and brain volumes among three age-stratified subgroups in typically developing controls

**Mean (SD)**	**Child**	**Adolescent**	**Adult**	**Statistics**
**Age range**	7-12	13-17	18-29	-
**Age, mean (SD)**	10.6 (1.4)	15.5 (1.6)	23.4 (3.0)	-
**Handedness, right (%)**	40 (93.0)	17 (94.4)	26 (89.7)	p = 0.807
**Intelligence Quotient (IQ)**				
Full-scale IQ	115.2 (10.0)	108.7 (11.4)	116.8 (10.2)	p = 0.032
Verbal IQ	115.4 (9.3)	110.2 (9.1)	116.1 (9.6)	p = 0.084
Performance IQ	112.6 (12.0)	105.8 (13.7)	116.4 (11.9)	p = 0.020
**Total gray matter volume (mm^3)**	814.2 (55.7)	793.3 (43.3)	734.2 (47.3)	p < 0.001
**Total white matter volume (mm^3)**	501.9 (37.5)	510.7 (40.6)	527.5 (37.0)	p = 0.023
**Total CSF volume (mm^3)**	324.6 (30.9)	341.3 (34.2)	351.2 (36.1)	p = 0.004
**Total brain volume (mm^3)**	1316.2 (90.9)	1304.1 (82.3)	1261.7 (81.4)	p = 0.033
**Total intracranial volume (mm^3)**	1640.8 (113.3)	1645.4 (103.7)	1612.9 (103.5)	p = 0.487

SD, standard deviation; CSF, cerebrospinal fluid; SD, standard deviation.
