# Supplementary material for: Regional brain volume differences between males with and without autism spectrum disorder are highly age-dependent
Source: Mol Autism. 2015 May 21;6:29. doi: 10.1186/s13229-015-0022-3 (PMC4455336; doi:10.1186/s13229-015-0022-3)
Supplement: Additional file 6: Table S6. — Global brain volume differences. [file 13229_2015_22_MOESM6_ESM.pdf]

Additional file 6: Table S6 Global brain volume differences

	Mean (SD)			
**Volume (mm^3)**	**ASD Group**	**TDC Group**	**$F_{(2,174)}$**	**p value**
**Total GM**	796.4 (66.5)	784.3 (61.6)	1.58	0.211
**Total WM**	520.4 (46.0)	511.9 (39.2)	1.73	0.190
**Total CSF**	352.5 (55.2)	336.5 (35.0)	5.28	0.023
**Total brain**	1316.8 (104.0)	1296.2 (88.7)	2.00	0.159
**Total Intracranial**	1669.3 (143.0)	1632.7 (108.1)	3.67	0.057

Abbreviations: ASD, autism spectrum disorder; TDC, typically developing control; GM, gray matter; WM, white matter; CSF, cerebrospinal fluid; SD, standard deviation.
