# Supplementary figures and images for: Regional brain volume differences between males with and without autism spectrum disorder are highly age-dependent
Source: Mol Autism. 2015 May 21;6:29. doi: 10.1186/s13229-015-0022-3 (PMC4455336; doi:10.1186/s13229-015-0022-3)

(A) Age distribution of ASD

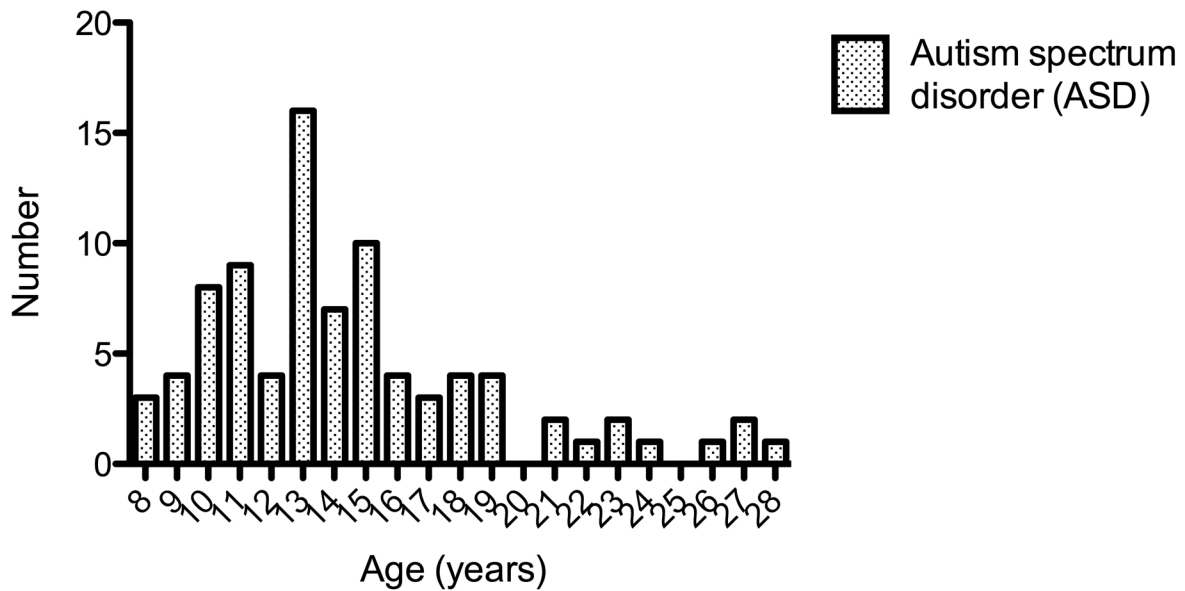

(B) Age distribution of TDC

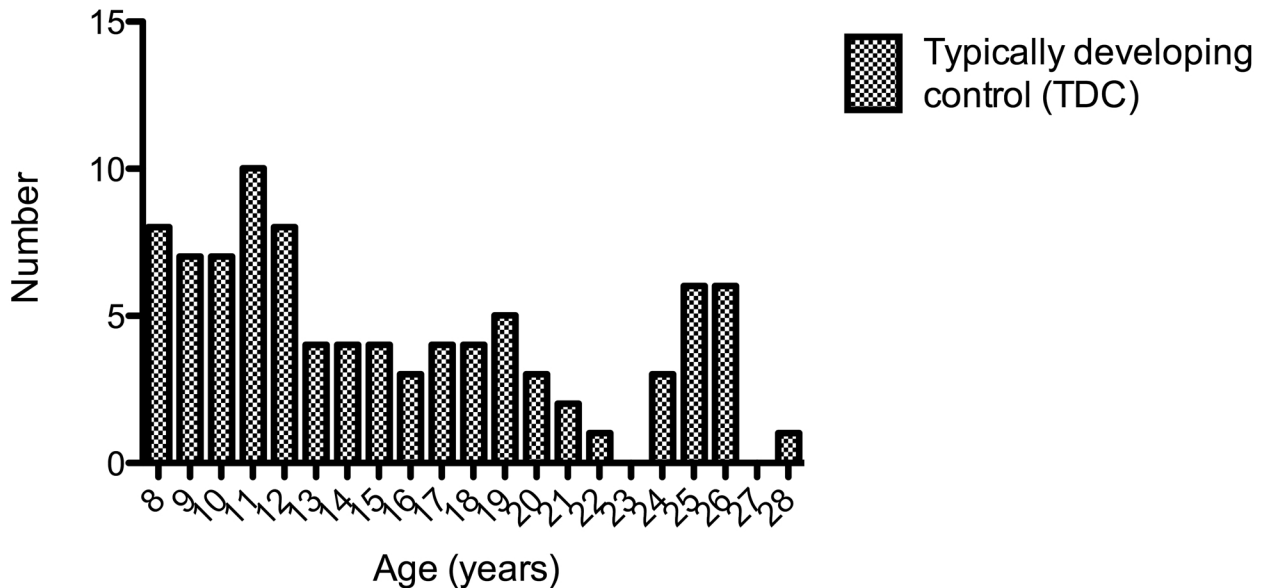

Supplement: Additional file 7: Figure S1. — Age distributions of participants with autism spectrum disorder and typically developing controls of the main analyses. [file 13229_2015_22_MOESM7_ESM.pdf]

(A) Age distribution of ASD (restricted age range)

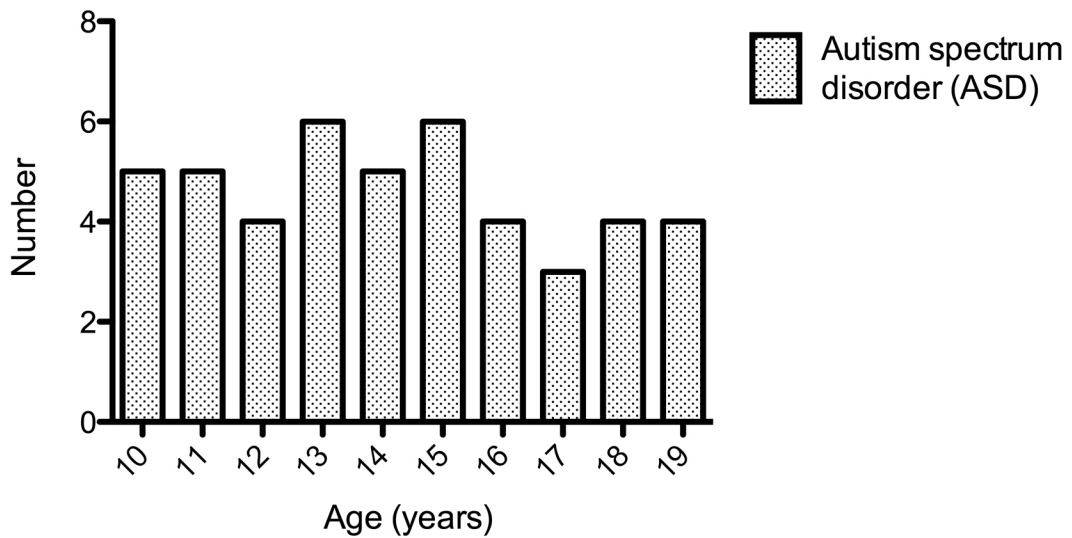

(B) Age distribution of TDC (restricted age range)

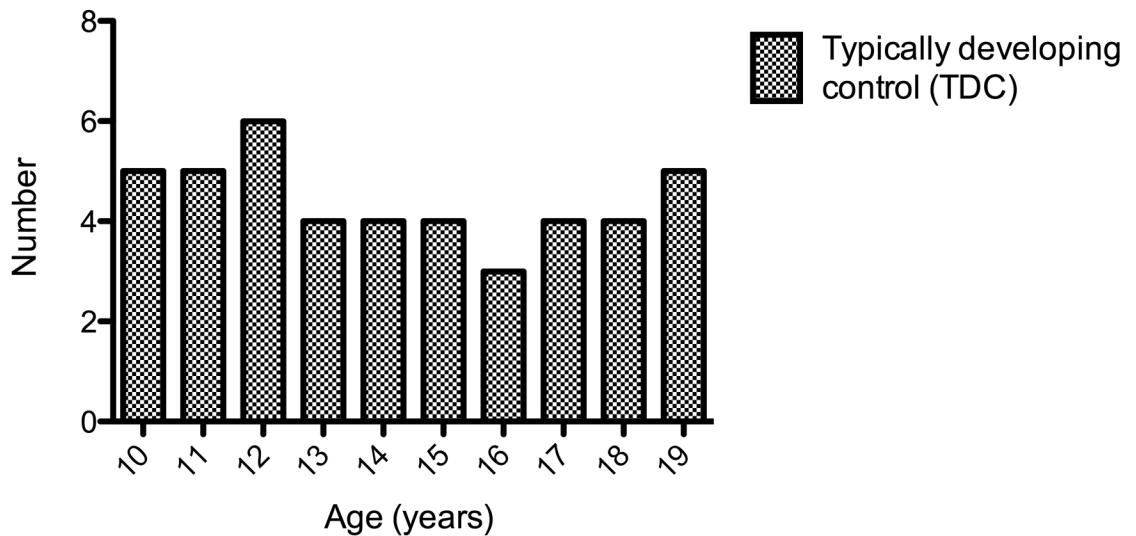

Supplement: Additional file 10: Figure S2. — Age distributions of participants with autism spectrum disorder and typically developing controls of the secondary analyses for the restrained age range. [file 13229_2015_22_MOESM10_ESM.pdf]
