# Supplementary material for: Regional brain volume differences between males with and without autism spectrum disorder are highly age-dependent
Source: Mol Autism. 2015 May 21;6:29. doi: 10.1186/s13229-015-0022-3 (PMC4455336; doi:10.1186/s13229-015-0022-3)
Supplement: Additional file 8: Table S7. — Significant differences in relative regional gray matter volume between participants with ASD and TDC participants, without controlling for full-scale IQ. [file 13229_2015_22_MOESM8_ESM.pdf]

Additional file 8: Table S7 Significant differences in relative regional gray matter volume between participants with ASD and TDC participants (without covarying IQ; the comorbidity status included as a categorical fixed-effect nuisance covariate)

Cluster	Region	BA	Hemisphere	MNI coordinates			T value	Cluster-level p value[a]	Cluster size (voxels)
				x	y	z			
**Model 1: ASD < TDC**									
Temporo-parieto-occipital junction cluster	Angular/middle occipital gyrus	39	L	-39	-75	32	4.27	0.035	476
	Precuneus/middle occipital gyrus	19	L	-34	-81	34	3.99		
	Precuneus/middle occipital gyrus	19	L	-27	-85	40	3.95		
**Model 2: ASD < TDC**									
Temporo-parieto-occipital junction cluster	Angular/middle occipital gyrus	39	L	-40	-75	32	4.28	0.030	488
	Precuneus/middle occipital gyrus	19	L	-34	-81	34	4.01		
	Precuneus/middle occipital gyrus	19	L	-27	-85	40	3.96		
**Model 2: ASD by age > TDC by age**									
Cuneus cluster	Cuneus	18	L	0	-88	18	5.43	<0.001	1957
	Cuneus	17	R	2	-82	12	4.53		
	Lingual gyrus	17	L	-8	-97	2	4.02		
Left anterior prefrontal cluster	Superior frontal gyrus	10	L	-18	71	-3	5.25	<0.001	1720
	Medial frontal gyrus	10	L	-6	71	-3	5.05		

		Middle frontal gyrus	10	L	-30	51	-8	5.00		
	Right anterior prefronal cluster	Superior frontal gyrus	10	R	21	71	4	4.54	<0.001	1211
		Medial frontal gyrus	10	R	6	71	-11	4.52		
		Medial frontal gyrus	10	R	5	68	7	4.45		
**Model 2: ASD by age < TDC by age**										
	Left cerebellum cluster	Crus I	…	L	-51	-58	-30	4.67	<0.001	1830
		Crus I	…	L	-46	-48	-32	4.39		
		Crus I	…	L	-42	-42	-34	4.04		
**Model 3: Child, ASD > TDC**										
	Limbic cluster			L	-7	2	-8	3.87	0.003	712
		Subcallosal gyrus	34	L	-12	5	-14	3.74		
		Extra-nuclear	…	R	0	-1	-6	3.72		
**Model 3: Child, ASD < TDC**										
	Anterior prefrontal cluster	Superior frontal gyrus	10	R	8	59	-11	4.70	0.001	787
		Superior frontal gyrus	10	L	-21	71	-3	4.43		
		Superior frontal gyrus	10	R	5	69	-12	3.99		
	Left Cuneus cluster	Cuneus	18	L	-27	-82	39	4.51	<0.001	1282
		Cuneus	18	L	-34	-81	34	4.32		
		Cuneus	18	L	-24	-88	18	3.88		
**Model 3: Adult, ASD > TDC**										
	Right dorsal medial prefrontal cluster	Superior frontal gyrus	10	R	11	57	36	5.80	<0.001	846
		Superior frontal gyrus	10	R	8	51	40	5.10		

	Superior frontal gyrus	10	R	8	69	19	4.65		
Right Cuneus cluster	Cuneus	18	R	2	-84	22	4.82	0.002	656
	Cuneus	18	R	0	-76	32	4.71		
	Cuneus	18	R	2	-87	14	4.68		
Left lateral prefrontal cluster	Superior frontal gyrus	10	L	-33	59	-3	4.28	0.007	539
	Middle frontal gyrus	10	L	-33	59	12	4.23		
	Middle frontal gyrus	10	L	-31	51	-8	4.07		

Abbreviations: ASD, autism spectrum disorder; TDC, typically developing control; BA, Brodmann area; L, left; R, right; ellipses, not applicable; MNI, Montreal Neurological Institute.

[a]Statistical threshold was all set at FWE-corrected cluster-level $p < 0.05$, with cluster-forming voxel-level $p < 0.005$
