# Supplementary material for: Regional brain volume differences between males with and without autism spectrum disorder are highly age-dependent
Source: Mol Autism. 2015 May 21;6:29. doi: 10.1186/s13229-015-0022-3 (PMC4455336; doi:10.1186/s13229-015-0022-3)
Supplement: Additional file 9: Table S8. — Significant differences in relative regional gray matter and white matter volume between participants with ASD and TDC participants in age-stratified analyses (model 3), only partialling out age effects as done in model 1. [file 13229_2015_22_MOESM9_ESM.pdf]

Additional file 9: Table S8 Significant differences in relative regional gray matter and white matter volume between participants with ASD and TDC participants in age-stratified analyses (Model 3), only partialling out age effects as done in Model 1

Cluster	Region	BA	Hemisphere	MNI coordinates			T value	Cluster-level p value[a]	Cluster size (voxels)
				x	y	z			
Gray Matter									
**Model 3: Child, ASD > TDC**									
Limbic cluster	Subcallosal gyrus	34	L	-12	5	-15	3.96	0.004	684
	Sub-lobar	…	R	5	5	-12	3.79		
	Sub-lobar	…	L	-7	2	-8	3.75		
**Model 3: Child, ASD < TDC**									
Right postcentral cluster	Postcentral gyrus	3	R	41	-34	63	4.31	0.023	512
	Postcentral gyrus	3	R	35	-25	69	4.21		
	Postcentral gyrus	3	R	30	-34	64	4.07		
Left parieto-occipital junction cluster	Precuneus	19	L	-27	-82	39	4.24	0.014	557
	Precuneus	19	L	-34	-82	34	4.06		
	Middle occipital gyrus	19	L	-32	-91	24	3.47		
**Model 3: Adult, ASD > TDC**									
Right dorsal medial prefrontal cluster	Superior frontal gyrus	9	R	12	56	34	5.37	<0.001	1186
	Superior frontal gyrus	10	R	8	51	42	4.87		
	Superior frontal gyrus	10	R	5	68	9	4.77		
Left anterior/ dorsal medial prefrontal cluster	Superior frontal gyrus	10	L	-7	56	1	4.81	0.006	555
	Superior frontal gyrus	10	L	-7	72	-3	4.74		
	Medial frontal gyrus	10	L	-10	71	6	4.24		
Left lateral prefrontal cluster	Middle frontal gyrus	10	L	-39	51	-17	4.44	0.033	416
	Superior frontal gyrus	10	L	-33	59	-3	4.40		

		Superior frontal gyrus	10	L	-27	57	-18	3.50		
	Cuneus cluster	Cuneus	18	R	2	-84	22	4.30	0.020	457
		Cuneus	18	…	0	-76	32	4.25		
		Cuneus	18	R	8	-91	14	4.11		
White Matter										
**Model 3: Adolescent, ASD > TDC**										
	Anterior corona radiata	Anterior cingulate gyrus	…	R	29	21	10	3.97	0.001	882
			…	R	11	29	-9	3.76		
			…	R	26	39	-5	3.52		

Abbreviations: ASD, autism spectrum disorder; TDC, typically developing control; BA, Brodmann area; L, left; R, right; ellipses, not applicable; MNI, Montreal Neurological Institute.

[a]Statistical threshold was all set at FWE-corrected cluster-level $p < 0.05$, with cluster-forming voxel-level $p < 0.005$.
