# Supplementary material for: Regional brain volume differences between males with and without autism spectrum disorder are highly age-dependent
Source: Mol Autism. 2015 May 21;6:29. doi: 10.1186/s13229-015-0022-3 (PMC4455336; doi:10.1186/s13229-015-0022-3)
Supplement: Additional file 11: Table S9. — Demographics of participants of restrained age range. [file 13229_2015_22_MOESM11_ESM.pdf]

Additional file 11: Table S9 Demographics of participants of restrained age-range

Mean (SD)	ASD Group (N = 46)	TDC Group (N = 44)	Statistics
**Age, mean (SD)**	14.67 (2.80)	14.50 (3.11)	p = 0.781
**Handedness, right (%)**	41 (89.1)	41 (93.2)	p = 0.714
**Intelligence Quotient (IQ)**			
Full-scale IQ	105.5 (15.3)	113.3 (10.0)	p = 0.006
Verbal IQ	106.1 (15.8)	114.1 (9.0)	p = 0.005
Performance IQ	104.2 (16.8)	110.2 (11.8)	p = 0.056
**Autism Diagnostic Interview-Revised**			
Social	21.1 (5.5)	…	…
Communication	15.3 (4.8)	…	…
Behavior	7.3 (2.7)	…	…
**Total gray matter volume (mm^3)**	793.9 (69.2)	802.0 (50.8)	p = 0.529
**Total white matter volume (mm^3)**	519.8 (48.1)	518.1 (39.1)	p = 0.847
**Total CSF volume (mm^3)**	340.3 (42.5)	340.4 (31.1)	p = 0.993
**Total brain volume (mm^3)**	1313.7 (110.3)	1320.1 (81.7)	p = 0.758
**Total intracranial volume (mm^3)**	1654.0 (140.6)	1660.4 (101.7)	p = 0.806

ASD, autism spectrum disorder; TDC, typically developing controls; CSF, cerebrospinal fluid; SD, standard deviation.
