# Supplementary material for: Regional brain volume differences between males with and without autism spectrum disorder are highly age-dependent
Source: Mol Autism. 2015 May 21;6:29. doi: 10.1186/s13229-015-0022-3 (PMC4455336; doi:10.1186/s13229-015-0022-3)
Supplement: Additional file 12: Table S10. — Significant differences in relative regional volume between participants with ASD and TDC participants of restrained age range. [file 13229_2015_22_MOESM12_ESM.pdf]

Additional file 12: Table S10 Significant differences in relative regional volume between participants with ASD and TDC participants of restrained age-range (10 to 19 years)

Cluster	Region	BA	Hemisphere	MNI coordinates			T value	Cluster-level p value	Cluster size (voxels)
				x	y	z			
**Gray matter**									
**Model 1: ASD < TDC**									
Lingual cluster	Lingual gyrus	29	L	-7	-49	2	3.80	0.043	441
	Precuneus	29	L	-6	-48	10	3.79		
	Lingual gyrus	…	R	2	-52	0	3.73		
**Model 2: ASD by age > TDC by age**									
Cuneus cluster	Cuneus	17	L	-4	-93	-2	4.59	< 0.001	1275
	Cuneus	18	…	0	-82	9	4.58		
	Cuneus	17	R	2	-75	15	3.98		
Cerebellum cluster	Cerebellar tonsil (Lobule VIII)	…	L	-24	-46	-58	4.34	0.002	723
	Cerebellar tonsil (Lobule VIII)	…	L	-34	-55	-48	3.92		
	Cerebellar tonsil (Lobule VIII)	…	L	-28	-42	-45	3.87		
**Model 2: ASD by age < TDC by age**									
Superior temporal cluster	Superior temporal gyrus	…	R	63	-33	9	4.27	0.015	533
	Middle temporal gyrus	…	R	69	-22	-5	3.91		
	Superior temporal gyrus	…	R	65	-10	9	3.78		
**White matter**									
**Model 2: ASD by age > TDC by age**									
Anterior limb of internal capsule	Globus pallidum	…	R	9	5	4	4.39	0.007	719
		…	R	12	8	-3	3.18		

…	R	2	2	6	3.08

ASD, autism spectrum disorder; TDC, typically developing control; BA, Brodmann area; L, left; R, right; MNI, Montreal Neurological Institute.
[a]Statistical threshold was all set at FWE-corrected cluster-level $p < 0.05$, with cluster-forming voxel-level $p < 0.005$.
